# Identifying the Sources of Tuberculosis in Young Children: A Multistate Investigation

**DOI:** 10.3201/eid0811.020419

**Published:** 2002-11

**Authors:** Sumi J. Sun, Diane E. Bennett, Jennifer Flood, Ann M. Loeffler, Steve Kammerer, Barbara A. Ellis

**Affiliations:** * California Department of Health Services, Berkeley, California, USA; † Centers for Disease Control and Prevention, Atlanta, Georgia, USA

**Keywords:** Mycobacterium tuberculosis, adolescent, child, children, molecular, restriction fragment length polymorphism, RFLP, source case, contact investigation

## Abstract

To better understand the molecular epidemiology of tuberculosis (TB) transmission for culture-confirmed patients <5 years of age, data were analyzed from a population-based study conducted in seven U.S. sites from 1996 to 2000. Mycobacterium tuberculosis isolates were genotyped with IS6110-based restriction fragment length polymorphism analysis and spoligotyping. Case-patient data were obtained from the Centers for Disease Control and Prevention’s national tuberculosis registry and health department records. Routine public health investigations conducted by local health departments identified suspected source patients for 57 (51%) of 111 culture-confirmed patients <5 years of age. For 8 (15%) of 52 culture-confirmed patients <5 years of age and their suspected source patients with complete genotyping results, genotypes suggested infection with different TB strains. Potential differences between sources for patients <5 years of age and source patients that transmitted TB to adolescent and adult patients were identified.

The occurrence of tuberculosis (TB) in children is an indicator of ongoing Mycobacterium tuberculosis transmission and of deficiencies in current public health efforts. In the United States, strategies to prevent childhood TB include identifying and promptly initiating treatment for adults with active TB to interrupt transmission (1–4). Since children have an increased risk for developing severe disease within weeks to months of infection, they are high priorities when identified as contacts to infectious patients (5,6).

For newly diagnosed TB in children, source-case investigations are conducted to ascertain the source of infection and to prevent ongoing transmission from infectious persons. Despite efforts by TB-control programs, suboptimal numbers of source patients are identified for children (7–12). Pinpointing the source of TB infection may be particularly challenging when numerous exposures exist, including contact with persons who reside outside the United States (13). Failure to find the true source patient may have treatment implications; decisions about the treatment regimen for children often hinge on the drug-susceptibility results of the suspected source patient because cultures from young children are often not available or attempts are not made to obtain these cultures (14).

The use of molecular analysis with conventional epidemiology has increased our understanding of TB transmission (15,16). In outbreaks and population-based studies, genotyping has been instrumental in identifying previously unsuspected connections among TB patients (17). Genotyping has also been used to evaluate epidemiologic links established through contact investigations. One report found that more than one quarter of index patients and their contacts who had TB and shared a household were infected with different TB strains, indicating that transmission did not occur between the household contacts (18).

In 1996, the Centers for Disease Control and Prevention (CDC) established the National Tuberculosis Genotyping and Surveillance Network (genotyping network) to conduct population-based genotyping in seven U.S. sentinel surveillance sites (19). During a 5-year period, the network collected information on culture-confirmed patients and their contacts with TB who were identified through routine public health investigations. Study sites also attempted to collect and genotype at least one M. tuberculosis isolate from each reported culture-confirmed case in the surveillance area.

To better understand the molecular epidemiology of TB transmission among young children (patients <5 years of age), data collected by the genotyping network were analyzed to report the frequency that suspected source patients were identified for young children, to examine the frequency and characteristics of source patients for young children, and to determine the proportion of isolates from young children and their identified source patients with discordant genotypes. We also investigated potential differences in the characteristics of source patients who transmitted TB to young children as compared to source patients who transmitted to adolescent and adult patients.

## Methods

### Collection of Epidemiologic Data

 A detailed description of study participants, population, and methodology is reported elsewhere (20). In brief, health department records were reviewed for all culture-confirmed patients who met the surveillance case definition (21) and were reported from the seven sites (Arkansas, California [six counties], Maryland, Massachusetts, Michigan, New Jersey, and Texas [four counties]) from January 1996 through December 2000. Contacts (of culture-confirmed patients in the sentinel areas) with active TB were identified through routine public health investigations, as defined by local contact and source-case investigation policies and practices at each study site. Source case investigations were undertaken for all patients <5 years of age. Two sites also routinely performed source case investigations on children <5 years of age. Information about epidemiologically related patients identified from public health investigations was gathered with a standardized data collection form that included the direction of transmission (i.e., whether the contact was a source patient or secondary patient in relation to the index patient or whether the direction of transmission was unknown), the relationship between patients (shared a household, nonhousehold friends or relatives, co-worker, or common source), and the exposure setting (correctional, school or day-care center, workplace, emergency shelter, group quarters, hospital, nursing home, other long-term care facility, or other setting). Data were entered into Epi Info version 6d (22) databases and routinely sent to CDC. State TB registry numbers for patients in the multisite genotyping network database were matched against the CDC’s national TB surveillance registry to obtain sociodemographic, behavioral, clinical, treatment, and drug-susceptibility information, which is routinely reported for all TB patients on the Report of Verified Patients of Tuberculosis (23). Project activities described in this paper were determined by CDC’s institutional review board to be exempt from full committee review since genotyping of isolates was considered a public health surveillance activity and all other data used in the analysis of this paper were previously collected.

### DNA Fingerprinting

 Genotyping of M. tuberculosis isolates was conducted in accordance with standardized study protocols (20). IS6110-based restriction fragment length polymorphism (RFLP) analysis was performed on all available isolates. Because low-copy numbers of IS6110 reduce test specificity, isolates containing six or fewer IS6110 copies were further analyzed by spacer oligonucleotide typing (spoligotyping) (24). Patients were determined to have concordant genotypes if their isolates contained seven or more IS6110 bands with identical patterns or six or fewer IS6110 bands with identical patterns and matching spoligotypes.

### Study Case Definitions

 Our investigation focused on culture-confirmed patients <5 years of age; TB in young children represents recent transmission, and source patient investigations are routinely conducted for this group. A source patient was defined as a confirmed TB patient who was identified by chart abstraction as the likely source of infection for another reported TB patient. A secondary patient was defined as a confirmed TB patient who was infected by an identifiable source. Epidemiologically related source patients and secondary patients identified through routine public health investigations were considered suspected patient pairs. Because some source patients transmitted TB to more than one secondary patient, the number of suspected patient pairs does not equal the number of source patients. A secondary patient, however, could have only one designated source patient.

 Genotypes for isolates from suspected patient pairs were compared, and patient pairs were categorized as 1) confirmed patient pairs, if isolates had concordant genotypes 2) refuted patient pairs, if isolates had discordant genotypes, and 3) undetermined patient pairs, if genotypes were unavailable for the patient pair.

### Data Analysis

 Data in the multisite genotyping network database were analyzed with SAS version 8.0 (25) and Epi Info version 6d (22) software packages. Patients were excluded from analysis when records were not available for review or lacked complete information from public health investigations, including three patients <5 years of age from one site, for whom source patients were not identified but who were entered into the database as the source for an adult case. Because young children are not typically considered to be infectious (26) and records for these patients were not available for further examination at the time of this analysis, information was determined to be incomplete for these patients.

 Univariate analysis was conducted to examine factors associated with the identification of source patients for young children and to investigate associations between key variables and the identification of refuted patient pairs. Differences in proportions were assessed with the chi-square statistic or 2-tailed Fisher exact test. Relative risks (RR) and 95% confidence intervals of point estimates were generated where appropriate. Differences in the means of continuous data were tested with the Wilcoxon rank-sum test when sample sizes were small. Unless otherwise noted, p values <0.05 were interpreted as statistically significant differences for all statistical tests.

 Genotypes of isolates from young children without a known source patient were matched against the genotyping network project database to find previously unidentified adult TB patient(s) whose genotype matched the child’s. Since the sentinel study sites represented geographically dispersed states that did not necessarily share a common border, genotype matches were limited to patients from the same site.

 To better describe the unique characteristics of patients who transmit TB to young children, source patients (in confirmed patient pairs) who transmitted TB to young children were compared with those who transmitted to adolescents or adult patients. Since source patients who infected children 5 years of age or older may be very similar to source patients who infected children newly born to 4 years of age, two different comparison groups were identified 1) source patients for all secondary patients ≥5 years of age and 2) source patients for secondary patients ≥15 years of age (excluding source patients that transmitted to children 5–14 years of age).

## Results

### Characteristics of Children with TB

 From 1996 to 2000, a total of 15,035 TB patients were reported from the seven sentinel surveillance sites; 11,923 (79%) were culture confirmed, and isolates from 10,752 (90%) culture-confirmed patients were genotyped. Of all patients in the study, 518 (3%) patients were <5 years of age. Culture was attempted in 270 (52%) patients <5 years of age, and 122 (45%) of these patients were culture confirmed. Isolates from 114 (93%) culture-confirmed children <5 years of age were genotyped.

 Texas and California sites reported 73 (60%) of the 122 culture-confirmed patients <5 years of age; the Michigan and New Jersey sites reported 18 patients each, and the remaining three sites reported ≤6 patients each. Most (65%) of the study patients were <2 years of age, and 49% were girls. Forty-three percent were black, non-Hispanic; 37% Hispanic; 15% Asian; 4% white, non-Hispanic, and 2% Native-American or Alaskan Native. Of the 11 foreign-born patients <5 years of age, 4 were from Mexico, 2 were from Kenya, and 5 were from other countries. Two thirds of the young children had pulmonary TB disease, 15% had extrapulmonary disease, and 20% had both pulmonary and extrapulmonary TB.

 With some notable exceptions, culture-confirmed patients <5 years of age had demographic or clinical characteristics similar to those of the 396 young children from the surveillance area who were either culture-negative or did not have a specimen collected for culture. The culture-confirmed group was more likely to be ≤1 year old (RR=1.94, p=<0.001); whereas white, non-Hispanic children (RR=0.41, p=0.02) and those treated only by private providers (RR=0.6, p=0.002) were underrepresented in the sample of culture-confirmed patients.

### Suspected Source Patients for Young Children

**Figure 1:**
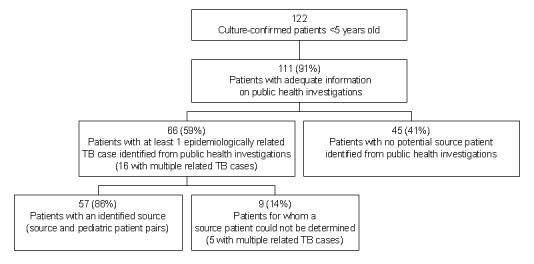
Results of public health investigations for culture-confirmed tuberculosis patients <5 years of age, 1996–2000.

Results of routine investigations used in identifying source patients for culture-confirmed children <5 years of age are presented in Figure 1. Health department records were unavailable or lacked sufficient information about investigations of 11 patients; these records were excluded. At least one epidemiologically related case was identified for 66 (59%) young children with culture-confirmed TB; 57 (86%) patients had a source patient designated, but a source could not be determined for the remaining 9 patients, although an epidemiologically related case was identified. For five of the nine patients, multiple epidemiologically related patients (ranging from 2–11 related patients) were identified.

 To examine factors associated with the identification of source patients for culture-confirmed children <5 years of age, we compared young children with a suspected source patient to patients with an unknown source of infection (Table 1). Children <2 years of age were more likely to have a source patient identified from routine public health investigations; however, source patients were less frequently found for foreign-born children. No other statistically significant differences were found. Drug-susceptibility patterns for isolates from young children with any drug resistance are detailed in Table 2.

**Table 1:** Factors associated with identifying source patients for culture-confirmed tuberculosis in children <5 years of age^a^

Characteristics	Suspected sources identified n=57 (%)		No suspected source identified n=45 (%)		Relative risk (95% CI)	p value
Age ≤2 yrs^b^	44	(77)	20	(45)	1.96 (1.23 to 3.12)	0.001
Female	30	(53)	21	(47)		NS
Race/ethnicity						NS
Black, non-Hispanic	26	(46)	19	(42)		
Hispanic	23	(40)	12	(27)		
Asian	5	(9)	11	(24)		
White, non-Hispanic	1	(2)	3	(7)		
Native American or Alaskan Native	2	(4)	0	(0)		
Foreign-born^c^	1	(2)	7	(16)	0.21 (0.03 to 1.31)	0.02
Type of disease						NS
Pulmonary only	40	(70)	30	(67)		
Extrapulmonary only	5	(9)	8	(18)		
Pulmonary and extrapulmonary	12	(21)	7	(16)		
Provider type^d^						NS
Health department	17	(31)	10	(23)		
Private provider	18	(33)	21	(49)		
Both	20	(36)	12	(28)		
Directly observed therapy^e^	46	(85)	28	(68)		NS
Drug-resistant isolate^f^	6	(11)	8	(16)		NS

^a^NS, not significant; CI, confidence interval. ^b^Age at start of treatment. Excludes one child whose date of treatment was unknown. ^c^Excludes one child whose birthplace was unknown. ^d^Excludes four children whose provider type was unknown. ^e^Compared to patients on self-administered therapy. ^f^Drug resistance on initial testing of isolate; resistance to at least one of the following: isoniazid, rifampin, ethambutol, pyrazinamide, streptomycin, and ethionamide. Testing results for one or more drugs could have been unknown or not done. Excludes two children for whom drug-susceptibility testing was not done.

**Table 2:** Drug-susceptibility patterns for isolates from culture-confirmed patients <5 years of age with and without a suspected source patient identified^a^

Suspected source patient identified	Source patient not identified
Ethionamide	Isoniazid
Streptomycin	Streptomycin
Streptomycin	Streptomycin
Isonazid, streptomycin	Pyrazinamide
Isonazid, streptomycin	Pyrazinamide
Isonazid, rifampin, ethambutol streptomycin	Isoniazid, streptomycin
	Isoniazid, rifampin
	Isoniazid, ethambutol, streptomycin

^a^Through routine public health investigations.

Table 3 lists characteristics of the 53 source patients identified from public health investigations. In 41 (72%) of 57 suspected patient pairs involving young children, the source patient lived in the child’s household. Of the 16 nonhousehold sources, 3 were babysitters, 4 were neighbors or visitors, 2 were relatives, and 1 attended the same church as the child’s family; the specific relationship was unknown for 6 patient pairs. Eight (15%) of the source patients resulted in disease in more than one young child (including culture-negative children and patients outside of the study population).

**Table 3:** Demographic, clinical, and risk characteristics of 53 source patients with tuberculosis (TB) identified from public health investigations^a^

Source patient characteristics	No. (%)
Age group, yrs	
15–24	11 (21)
25–44	28 (53)
45–64	10 (19)
65+	4 (8)
Female	24 (45)
Race or ethnicity	
Black, non-Hispanic	22 (42)
Hispanic	24 (45)
Asian	5 (9)
Native American or Alaskan Native	2 (4)
Foreign-born^b^	27 (51)
Bacteriologic results, sputum	
Smear positive/culture positive	42 (79)
Smear positive/culture negative	1 (2)
Smear negative/culture positive	8 (15)
Smear not done/culture not done	2 (4)
Cavitary chest radiograph^c^	33 (63)
Provider type	
Health department	31 (58)
Private provider	11 (11)
Both	11 (11)
Directly observed therapy^d^	47 (71)
Previous diagnosis of TB	5 (9)
Drug-resistant isolate^e^	5 (9)

^a^Three source patients were identified as the source of infection for more than one culture-confirmed patient who was <5 years of age in the sentinel study population; two source patients transmitted to two children, and one transmitted to three children. ^b^Country of origin was Mexico for 14 (26%) of the foreign-born patients. ^c^Results unknown for one patient. ^d^Compared to patients on self-administered therapy. ^e^Drug resistance on initial testing of isolate; resistance to at least one of the following: isoniazid, rifampin, ethambutol, pyrazinamide, streptomycin, and ethionamide. Testing results for one or more drugs could have been unknown or not done. Excludes one source patient for whom drug susceptibility testing was not done.

### Molecular Fingerprint Data

**Figure 2:**
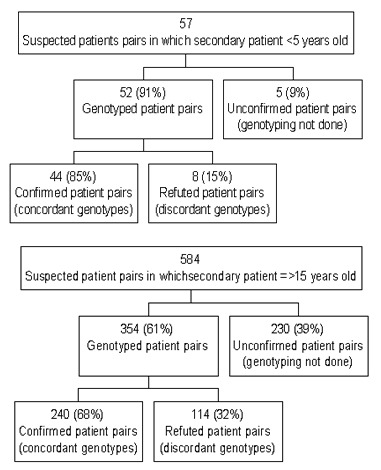
Comparison of genotypes for isolates from suspected source and secondary patient pairs identified through public health investigations, 1996–2000. Culture-confirmed children <5 years of age and their sources versus culture-confirmed patients ≥15 years of age and their sources.

Of the 57 culture-confirmed patients <5 years of age for whom a source patient was identified, 91% (52) had genotyping results for both the young child and the suspected source patient (Figure 2). Forty-four (85%) of 52 suspected patient pairs had concordant genotypes, and 8 (15%) of 52 had discordant genotypes. Young children in refuted patient pairs were more likely to be older than those in confirmed patient pairs (Table 4). No association between gender, ethnicity, or foreign-born status of patients and the identification of refuted patient pairs was found. Nearly three quarters (37 of 52) of suspected patient pairs lived in the same household; however, 5 (14%) of these patient pairs had discordant genotypes. Suspected patient pairs with differing drug-susceptibilities were not associated with discordant genotypes; all three patient pairs with differing drug resistance patterns had concordant genotypes (Table 5).

**Table 4:** Characteristics of refuted and confirmed patients pairs^a^

	Refuted patient pairs n=8 (%)	Confirmed patient pairs n=44 (%)
Characteristics of young children		
Mean age, months^b^	16	13
Female	4 (50)	23 (52)
Race/ethnicity		
Black, non-Hispanic	5 (63)	20 (45)
Hispanic	3 (38)	16 (36)
Asian	0 (0)	5 (11)
White, non-Hispanic	0 (0)	1 (2)
Native American/Alaskan Native	0 (0)	2 (5)
Foreign-born	1 (13)	0 (0)
Source patient characteristics		
Mean age, yrs	25	31
Female	6 (75)	18 (41)
Race or ethnicity		
Black, non-Hispanic	5 (63)	20 (45)
Hispanic	3 (38)	17 (39)
Asian	0 (0)	5 (11)
Native American or Alaskan Native	0 (0)	2 (5)
Foreign-born	3 (38)	20 (45)
Case patient characteristics		
Shared household	5 (63)	32 (73)
Discordant drug susceptibilities^c^	0 (0)	3 (7)
Different race/ethnicity	0 (0)	1 (2)

^a^For tuberculosis patients <5 years of age and their suspected source patients. Refuted patient pairs are suspected patient pairs with discordant genotypes; confirmed patient pairs are suspected patient pairs with concordant genotypes. ^b^Wilcoxon rank-sum test: p=0.03. ^c^Excludes two patient pairs in which the children had drug-resistant *Mycobacterium tuberculosis* strains (streptomycin and ethambutol resistance, respectively), but susceptibility results were not done for the identified source patient.

**Table 5:** Comparison of drug-susceptibility and genotyping results for isolates of suspected patient pairs^a^ with any drug resistance

Source-patient isolate	Secondary patient isolate (children <5 years of age)	Drug-susceptibility comparison (patient pairs)	Genotype comparison (patient pairs)
Isoniazid	Isoniazid, streptomycin	Discordant	Concordant
Isoniazid, rifampin	Isoniazid, rifampin, ethambutol, streptomycin	Discordant	Concordant
Streptomycin	Pan-susceptible	Discordant	Concordant
Streptomycin	Streptomycin	Concordant	Concordant
Isoniazid, streptomycin	Isoniazid, streptomycin	Concordant	Concordant
Pan-susceptible^b^	Ethionamide	Undetermined^c^	Concordant
Not done	Streptomycin	Undetermined	Undetermined

^a^Tuberculosis patients <5 years of age and their suspected source patients. ^b^Isolate from source patient was not tested for ethionamide resistance. ^c^Undetermined, results unknown for one or both patients.

For the nine young patients who had at least one epidemiologically related patient identified by public health investigations but for whom the source patient could not be determined, genotyping patterns from the isolates of the epidemiologically related cases and the young child were identical, almost without exception. The only two discordant genotypes were in young children with a single related case, not among the five young children with multiple related patients.

Genotyping also identified patients in the local surveillance site who had the same genotype as young children without an identified source patient. Isolates were genotyped from 40 of 45 patients <5 years of age without a known source patient. Of these genotyped isolates, 23 (58%) matched the strain from at least l adult pulmonary TB case in the local surveillance site. For most young children (13 [57%] of 23) without an identified source patient, at least 5 adult pulmonary TB patients with genotypes matching the child’s were identified. We found a wide range in the number of adult patients (2–128) with genotypes matching the genotypes of these young children.

### Confirmed Source Patients for Children, Adolescents, and Adults

To better characterize the unique attributes of patients who transmit TB to young children, characteristics of their source patients (in confirmed patient pairs) were compared with those for adults and adolescents. No significant differences were found when the comparison group for this analysis consisted of all sources to secondary patients ≥5 years of age or when the comparison group was limited to sources to secondary patients ≥15 years of age. The results of the latter comparison are presented. More than 60% (354 of 584) of the suspected patient pairs in which the secondary patient was not a child were genotyped, and 240 (68%) of these patient pairs had concordant genotypes (Figure 2). The likelihood of identifying patient pairs with discordant genotypes was more than two times higher among suspected patient pairs involving secondary patients ≥15 years of age than for those involving young children (32% vs. 15% discordant genotypes) (RR=2.09, p=0.01).

 Univariate associations between source patient characteristics and transmission to young children were assessed (Table 6). Although the mean age for sources to secondary patients <5 years of age was slightly lower than the mean age of sources to the comparison group, these differences were not significant (p=0.06; Wilcoxon test). For this population, confirmed source patients to young children were more likely to be foreign-born (p=0.02), Hispanic (p<0.001), a household member (p<0.001), and not receiving directly observed therapy (p<0.01) as compared with sources for adolescents and adults.

**Table 6:** Characteristics of source patients in confirmed patient pairs^a^

Source patient characteristics	Confirmed sources for secondary patients <5 yrs of age n=44 (%)	Confirmed sources for secondary patients ≥15 ys of age n=240 (%)
Mean age, yrs	31	38
Female	18 (41)	103 (43)
Race/ethnicity^b^		
Black, non-Hispanic	20 (45)	148 (62)
Hispanic	17 (39)	23 (10)
Asian	5 (11)	19 (8)
White, non-Hispanic	0	48 (20)
Native American or Alaskan Native	2 (5)	2 (1)
Foreign-born^c^	20 (45)	56 (23)
Bacteriologic results, sputum^d^		
Smear positive/culture positive	34 (81)	200 (85)
Smear negative/culture positive	8 (19)	33 (14)
Smear negative/culture negative	0	1 (<1)
Cavitary chest radiograph^e^	27 (64)	125 (53)
Provider type^f^		
Health department	25 (57)	141 (60)
Private provider	9 (20)	43 (18)
Both	10 (23)	52 (22)
Directly observed therapy ^b,g^	30 (68)	198 (84)
Previous diagnosis of TB^h^	6 (14)	27 (11)
Shared household with secondary case ^b,i^	32 (91)	116 (50)

^a^Confirmed patient pairs include source patients who transmitted TB to young children and source patients who transmitted TB to adolescent and adult patients. ^b^Chi-square statistic, p<0.05. ^c^Country of origin unknown for one patient. ^d^Excludes eight patients in whom either the culture or smear was not done. ^e^Chest radiograph results unknown for two patients. ^f^Provider type unknown for four patients. ^g^Compared to patients on self-administered therapy only. Directly observed therapy status unknown for six patients. ^h^History of TB unknown for two patients. ^i^Relationship to secondary case unknown for 19 patients.

## Discussion

Despite the continued decline in the number of TB patients in the United States, ongoing TB transmission persists in many communities. For public health agencies, TB in young children signals recent transmission and missed opportunities for TB prevention. In this investigation, molecular tools were used in conjunction with information from conventional public health investigations to better understand issues related to the identification of source patients for young children.

 In this multisite study, 57 (51%) of 111 culture-confirmed patients <5 years of age had a source patient identified by routine investigations. Although this finding is comparable to the frequency of source patient identification reported for other subpopulations of children with TB (8,10–11), the finding may be lower than anticipated for a sample of young children predominantly born in the United States. Children <2 years of age and those born in the United States were more likely to have a source patient found than children without these characteristics. These results corroborated findings from a study of children <5 years of age with TB in California, which demonstrated that the source of infection is more likely to be identified for children who were found in a contact investigation, born in the United States, <1 year of age, or black (9).

 Children <5 years of age with an unknown source of infection composed a substantial proportion of the study population (41%), a finding that underscores shortcomings in identifying all contacts of infectious patients. While molecular data alone are not enough to prove recent transmission, the presence of infectious TB patients in the community who share the same strain with a young child without a known source suggests the possibility of casual transmission. Other impediments in identifying source patients may include barriers in completing contact investigations, delays in evaluation, and problems in identifying source patients who reside outside the health department’s jurisdiction (27). Eighty-four percent of young children without a source patient in this study were born in the United States; this observation is likely to underestimate the contribution of the global TB epidemic, because TB surveillance systems in the United States do not routinely monitor the birthplace or travel history of parents or guardians, factors previously identified as significant predictors for pediatric TB (28,29).

 Of particular concern is the finding that 16 (14%) of 111 culture-confirmed young children had more than one epidemiologically related TB source identified. This finding indicates that a substantial number of children have multiple TB exposures that need to be carefully assessed. For most, the source of infection was ascertained and later confirmed by genotyping analysis. When multiple epidemiologically related patients existed, but none was identified as the source patient, genotyping analysis did not provide added benefit since the related patients were more likely to have the same genotype.

 Clinicians and TB-control programs often rely on the drug-susceptibility results of the suspected source patient to guide the treatment of the child since specimens for culture are not frequently collected from children (14). Previous studies by Steiner et al. reported 93% to 96% concordance of drug-susceptibility patterns of TB isolates from children <15 years of age and their source (30,31), comparable to the 93% drug-susceptibility concordance among suspected patient pairs in our study population. All suspected patient pairs with discordant drug-susceptibility results were among patient pairs with concordant genotypes, indicating the value of drug-susceptibility results in young children, even when genotyping results are known to the local health department.

 The high frequency (85%) of concordant genotypes among young children and their source patients represents good news for public health agencies; when a potential source of infection was identified in this population, it was most often accurate. However, for as many as 15%, the true source was not identified and presumably could have contributed to the further spread of disease in the community. Because young children may have more limited opportunities for exposure than older children, we anticipated that the frequency of confirmed patient pairs would be associated with young age. We also speculated that foreign-born children, especially those from high TB-prevalence areas, might have an increased risk of being involved in a refuted patient-pair. These children might have had multiple opportunities for exposure to active TB before entering the United States, which may increase the possibility that the source of infection could have been someone other than the suspected source patient. However, this potential association could not be assessed because our sample of foreign-born children with culture-confirmed TB was small.

 The increased likelihood of concordant genotypes among suspected patient pairs involving young children as compared with suspected patient pairs that did not include children (85% vs. 68%) may be explained by a number of factors, including the greater number of casual contacts with whom adults interact, biases in the case-finding practices for these groups, and potential for coincidental reactivation of a latent TB infection in older patient pairs. Source patients who transmitted TB to young children were more likely to be Hispanic, foreign-born, a household member, and not receiving directly observed therapy as compared to sources for adolescents or adults. The latter may indicate nonadherence of source patients to drug treatment and corroborates an observation by Kimerling et al. (32). However, additional data are needed to determine the confounding factors, including site-to-site variance, that may affect which source patients receive directly observed therapy, as discussed in the limitations that follow.

A key limitation in this study was the inability to assess the effect of potential confounding factors, such as differences in case-finding methods (i.e., if patients were identified through contact investigation, source patient investigations, or screening activities) on the outcome of interest (i.e., identification of source patients or confirmed patient pairs). These data represented the sites’ routine public health practices and policies, since uniform policies for public health investigations were not instituted, and potential systematic variances across sites were not ascertained by the project. In addition, analysis of epidemiologic investigations for infectious patients in the community who shared the same TB strain as the young child but were not identified from public health investigators was outside the scope of this paper. A follow-up investigation to find epidemiologic connections among patients currently linked by genotyping results alone may provide important data regarding potential missed opportunities in this group. Finally, the predictive value of a discordant genotype result is not yet known. Although study protocols instituted quality-control measures across genotyping laboratories, a subset of isolates from suspected patient pairs who were determined to have discordant genotypes might include TB strains that are potentially the same. Thus, the proportion of discordance observed in this study may represent an overestimate of the actual frequency of suspected patient pairs with discordant genotypes.

This study highlights the challenges in identifying the sources of infection for culture-confirmed children under 5 years of age and potential weakness in our current TB-control and prevention practices in this population. Although contact and source patient investigations are central to any TB-control strategy, the usefulness of these activities in identifying the true source of infection in young children has not been previously evaluated for a large population of children by using molecular methods. While indicating a high degree of concordance between genotypes from young children and their identified sources, genotyping analysis also refuted some source patients and pointed to other potential sources in the community who were previously unsuspected. Further assessment of shortcomings in current methods to prevent transmission to children and to identify their source of infection is warranted to ultimately eliminate TB in young children in the United States.

## References

[1] Control of tuberculosis in the United States. American Thoracic Society. Am Rev Respir Dis. 1992;146:1623–33. 1456588 10.1164/ajrccm/146.6.1623

[2] Perry S, Starke JR. Adherence to prescribed treatment and public health aspects of tuberculosis in children. Semin Pediatr Infect Dis. 1993;4:291–8.

[3] Centers for Disease Control and Prevention. Screening for tuberculosis and tuberculosis infection in high-risk populations: recommendations of the Advisory Council for the Elimination of Tuberculosis. MMWR Morb Mortal Wkly Rep. 1995;44:19–34. 7565540

[4] Centers for Disease Control and Prevention. Essential components of a tuberculosis prevention and control program: recommendations of the Advisory Council for the Elimination of Tuberculosis. MMWR Morb Mortal Wkly Rep. 1995;44:1–16. 7565539

[5] Fujiwara PI, ed. Clinical policies and protocols. New York: New York City Department of Health, Bureau of Tuberculosis Control; 1999. p. 93–7.

[6] Marshman FC, ed. Prevention and control of pediatric tuberculosis in New York City: recommendation for the expert advisory panel. New York: New York City Department of Health; 1995. p. 18–9.

[7] Driver CR, Luallen JJ, Good WE, Valway SE, Frieden TR, Onorato IM. Tuberculosis in children younger than five years of age: New York City. Pediatr Infect Dis J. 1995;14:112–7. 7746692 10.1097/00006454-199502000-00006

[8] Gessner BD. Incidence rates, clinical features, and case identification of pediatric tuberculosis in Alaska. Int J Tuberc Lung Dis. 1998;2:378–83. 9613633

[9] Lobato MN, Mohle-Boetani JC, Royce SE. Missed opportunities for preventing tuberculosis among children younger than five years of age. Pediatrics. 2000;106:E75. 10.1542/peds.106.6.e75 11099618

[10] Lobato MN, Loeffler AM, Furst K, Cole B, Hopewell PC. Detection of *Mycobacterium tuberculosis* in gastric aspirates collected from children: hospitalization is not necessary. Pediatrics. 1998;102:E40. 10.1542/peds.102.4.e40 9755277

[11] Moss W. Tuberculosis in the home: contact history and childhood tuberculosis in central Harlem. Clin Pediatr (Phila). 1998;37:753–5. 10.1177/000992289803701208 9864652

[12] Watchi R, Kahlstrom E, Vachon LA, Barnes PF. Pediatric tuberculosis: clinical presentation and contact investigation at an urban medical center. Respiration. 1998;65:192–4. 10.1159/000029258 9670300

[13] Sullam PM, Slutkin G, Hopewell PC. The benefits of evaluating close associates of child tuberculin reactors from a high prevalence group. Am J Public Health. 1986;76:1109–11. 10.2105/AJPH.76.9.1109 3740335 PMC1646577

[14] Al-Dossary F, Ong L, Correa A, Starke JR. Treatment of childhood tuberculosis with a six month directly observed regimen of only two weeks daily therapy. Pediatr Infect Dis J. 2002;21:91–2. 10.1097/00006454-200202000-00002 11840073

[15] Jasmer RM, Hahn JA, Small PM, Daley CL, Behr MA, Moss AR, A molecular epidemiologic analysis of tuberculosis trends in San Francisco, 1991–1997. Ann Intern Med. 1999;130:971–8. 10383367 10.7326/0003-4819-130-12-199906150-00004

[16] Behr MA, Warren SA, Salamon H, Hopewell PC, Ponce de Leon A, Daley CL, Transmission of *Mycobacterium tuberculosis* from patients smear-negative for acid-fast bacilli. Lancet. 1999;353:444–9. 10.1016/S0140-6736(98)03406-0 9989714

[17] Chin DP, Crane CM, Diul MY, Sun SJ, Agraz R, Taylor S, Spread *of Mycobacterium tuberculosis* in a community implementing recommended elements of tuberculosis control. JAMA. 2000;283:2968–74. 10.1001/jama.283.22.2968 10865275

[18] Behr MA, Hopewell PC, Paz EA, Kawamura LM, Schecter GF, Small PM. Predictive value of contact investigation for identifying recent transmission of *Mycobacterium tuberculosis.* Am J Respir Crit Care Med. 1998;158:465–9. 9700122 10.1164/ajrccm.158.2.9801062

[19] Castro KG, Jaffe HW. Rationale and methods for the National Tuberculosis Genotyping and Surveillance Network. Emerg Infect Dis. 2002;8:1188–91. 12453341 10.3201/eid0811.020408 PMC2738540

[20] Crawford JT, Braden CR, Schable BA. National tuberculosis genotyping and surveillance network: designs and methods. Emerg Infect Dis. 2002;8:1192–6. 12453342 10.3201/eid0811.020296 PMC2737808

[21] Centers for Disease Control and Prevention. Case definitions for public health surveillance. MMWR Morb Mortal Wkly Rep. 1990;39:39–40.

[22] Epi Info. a word processing, database, and statistics program [computer program]. Version 6. Atlanta: Centers for Disease Control and Prevention; 1994.

[23] Centers for Disease Control and Prevention. Tuberculosis information management system (TIMS) user’s guide. Surveillance appendix 1. Atlanta: U.S. Department of Health Services, Public Health Service; 1999.

[24] Bauer J, Andersen AB, Kremer K, Miorner H. Usefulness of spoligotyping to discriminate IS6110 low-copy-number *Mycobacterium tuberculosis* complex strains cultured in Denmark. J Clin Microbiol. 1999;37:2602–6. 10405409 10.1128/jcm.37.8.2602-2606.1999 PMC85294

[25] SAS/STAT user’s guide. Cary (NC): SAS Institute Inc.; 2000.

[26] A statement of the Scientific Committee of the IUATLD. Tuberculosis in children: guidelines for diagnosis, prevention, and treatment. Bull Int Union Tuberc Lung Dis. 1991;66:114–9. 1859945

[27] Besser RE, Pakiz B, Schulte JM, Alvarado S, Zell ER, Kenyon TA, Risk factors for positive mantoux tuberculin skin tests in children in San Diego, California: evidence for boosting and possible foodborne transmission. Pediatrics.;108:305–10. 11483792 10.1542/peds.108.2.305

[28] Kenyon TA, Driver C, Haas E, Valway SE, Moser KS, Onorato IM. Immigration and tuberculosis among children on the United States-Mexico border, County of San Diego, California. Pediatrics. 1999;104:E8. 10.1542/peds.104.1.e8 10390294

[29] Saiman L, San Gabriel P, Schulte J, Vargas MP, Kenyon T, Onorato I. Risk factors for latent tuberculosis infection among children in New York City. Pediatrics. 2001;107:999–1003. 10.1542/peds.107.5.999 11331677

[30] Steiner M, Zimmerman R, Park BH, Shirali SR, Schmidt IT. Primary tuberculosis in children: II. Correlation of susceptibility patterns of *Mycobacterium tuberculosis* isolated from children with those isolated from source cases as an index of drug resistant infection in a community. Am Rev Respir Dis. 1968;98:201–9. 4970045 10.1164/arrd.1968.98.2.201

[31] Steiner P, Rao M, Mitchell M, Steiner M. Primary drug-resistant tuberculosis in children. Correlation of drug-susceptibility patterns of matched patient and source case strains of *Mycobacterium tuberculosis.* Am J Dis Child. 1985;139:780–2. 3927707 10.1001/archpedi.1985.02140100042023

[32] Kimerling ME, Vaughn ES, Dunlap NE. Childhood tuberculosis in Alabama: epidemiology of disease and indicators of program effectiveness, 1983 to 1993. Pediatr Infect Dis J. 1995;14:678–84. 10.1097/00006454-199508000-00006 8532425

